# Functional hydrogels for the repair and regeneration of tissue defects

**DOI:** 10.3389/fbioe.2023.1190171

**Published:** 2023-05-16

**Authors:** Xinlin Li, Mengfei Xu, Zhaoli Geng, Yi Liu

**Affiliations:** Department of Orthodontics, School and Hospital of Stomatology, Cheeloo College of Medicine, Shandong University and Shandong Key Laboratory of Oral Tissue Regeneration and Shandong Engineering Laboratory for Dental Materials and Oral Tissue Regeneration and Shandong Provincial Clinical Research Center for Oral Diseases, Jinan, China

**Keywords:** functional hydrogels, tissue defects, bone, cartilage, skin, muscle, nerve

## Abstract

Tissue defects can be accompanied by functional impairments that affect the health and quality of life of patients. Hydrogels are three-dimensional (3D) hydrophilic polymer networks that can be used as bionic functional tissues to fill or repair damaged tissue as a promising therapeutic strategy in the field of tissue engineering and regenerative medicine. This paper summarises and discusses four outstanding advantages of hydrogels and their applications and advances in the repair and regeneration of tissue defects. First, hydrogels have physicochemical properties similar to the extracellular matrix of natural tissues, providing a good microenvironment for cell proliferation, migration and differentiation. Second, hydrogels have excellent shape adaptation and tissue adhesion properties, allowing them to be applied to a wide range of irregularly shaped tissue defects and to adhere well to the defect for sustained and efficient repair function. Third, the hydrogel is an intelligent delivery system capable of releasing therapeutic agents on demand. Hydrogels are capable of delivering therapeutic reagents and releasing therapeutic substances with temporal and spatial precision depending on the site and state of the defect. Fourth, hydrogels are self-healing and can maintain their integrity when damaged. We then describe the application and research progress of functional hydrogels in the repair and regeneration of defects in bone, cartilage, skin, muscle and nerve tissues. Finally, we discuss the challenges faced by hydrogels in the field of tissue regeneration and provide an outlook on their future trends.

## 1 Introduction

Tissue defects can be accompanied by functional impairment, affecting the patient’s health and quality of life. Damage to tissues such as bone, cartilage and muscle, which are essential for force production, body movement, postural support and maintenance of internal organ function, can lead to impairment of body movement and organ function (Gilbert-Honick and Grayson, 2020; Heng et al., 2023). The skin tissue is the barrier between the body and the external environment, which plays an important role in protecting internal organs, preventing the outflow of tissue fluid, sensing external stimuli, regulating body temperature and resisting friction and infection. Damage to skin tissue may cause disfigurement, pain, infection, dehydration, disability and death to the patient (Shu et al., 2021). Meanwhile, neurological deficits such as stroke, Parkinson’s disease, Alzheimer’s disease, Huntington’s disease, spinal cord injury and other neurological deficits may cause speech difficulties, cognitive impairment, memory loss, dementia, depression, disability and death (Jagaran and Singh, 2021). Therefore, it is essential to treat tissue defects and restore function in a timely manner.

The most common method to facilitate the repair and regeneration of severe tissue defects is to perform transplantation (autograft, allograft and xenograft). However, transplantation is greatly limited by the complexity of the procedure, immune rejection, lack of donor tissue and potential for dysfunction in the donor area (Carrier et al., 2022; Sykes and Sachs, 2022). The use of 3D biodegradable scaffolds as bionic functional tissues to fill or repair damaged tissues is a promising therapeutic strategy in the field of tissue engineering and regenerative medicine (He et al., 2020; Qi et al., 2021). The great advances in tissue engineering and regenerative medicine recently have been due in large part to the continued development and rapid growth of biomaterials such as hydrogels.

Hydrogels are essentially three-dimensional (3D) hydrophilic polymer networks that can remain structurally undamaged and insoluble while containing more than 90% water, making them particularly suitable for applications in cell culture and tissue engineering (Rastogi and Kandasubramanian, 2019; Vera et al., 2021). Moreover, hydrogels can be adapted to meet specific requirements under different conditions by adjusting their material composition, concentration, synthesis strategy, manufacturing process and cross-linking method. (Guimarães et al., 2021). As a kind of promising biocompatible material, hydrogels have outstanding advantages in the repair and regeneration of tissue defects. The physicochemical properties of hydrogels, such as mechanical strength, degradability and pore size, are similar to those of the extracellular matrix of natural tissues and can provide a good microenvironment for cell proliferation and differentiation during tissue regeneration. In the early stages of tissue repair, the hydrogel provides suitable mechanical support for the growth of new tissue. As the repair progresses, the hydrogel gradually degrades, providing space for the new tissue to repair (Yang and Chun, 2020; Lee et al., 2021). The excellent rheological and viscoelastic properties of hydrogels give them excellent shape adaptation and tissue adhesion. As a result, hydrogels can be applied to a wide range of irregularly shaped tissue defects and adhere well to the defect for efficient and sustainable restorative function (Yuan et al., 2021; Wang S. et al., 2022). The responsive hydrogel with its porous structure can not only load various therapeutic substances but also release them with temporal and spatial precision in response to internal and external environmental stimuli such as light (especially near-infrared light), pH, temperature, redox potential and magnetism (Yang and Chun, 2020; Li D. et al., 2022). Many hydrogels are polymer networks based on reversible noncovalent interactions or chemical bonding, which can perform self-healing to maintain their integrity when subjected to external tensions, mechanical stresses and other damaging factors (Zheng B. S. et al., 2022; Cai et al., 2022).

**FIGURE 1:**
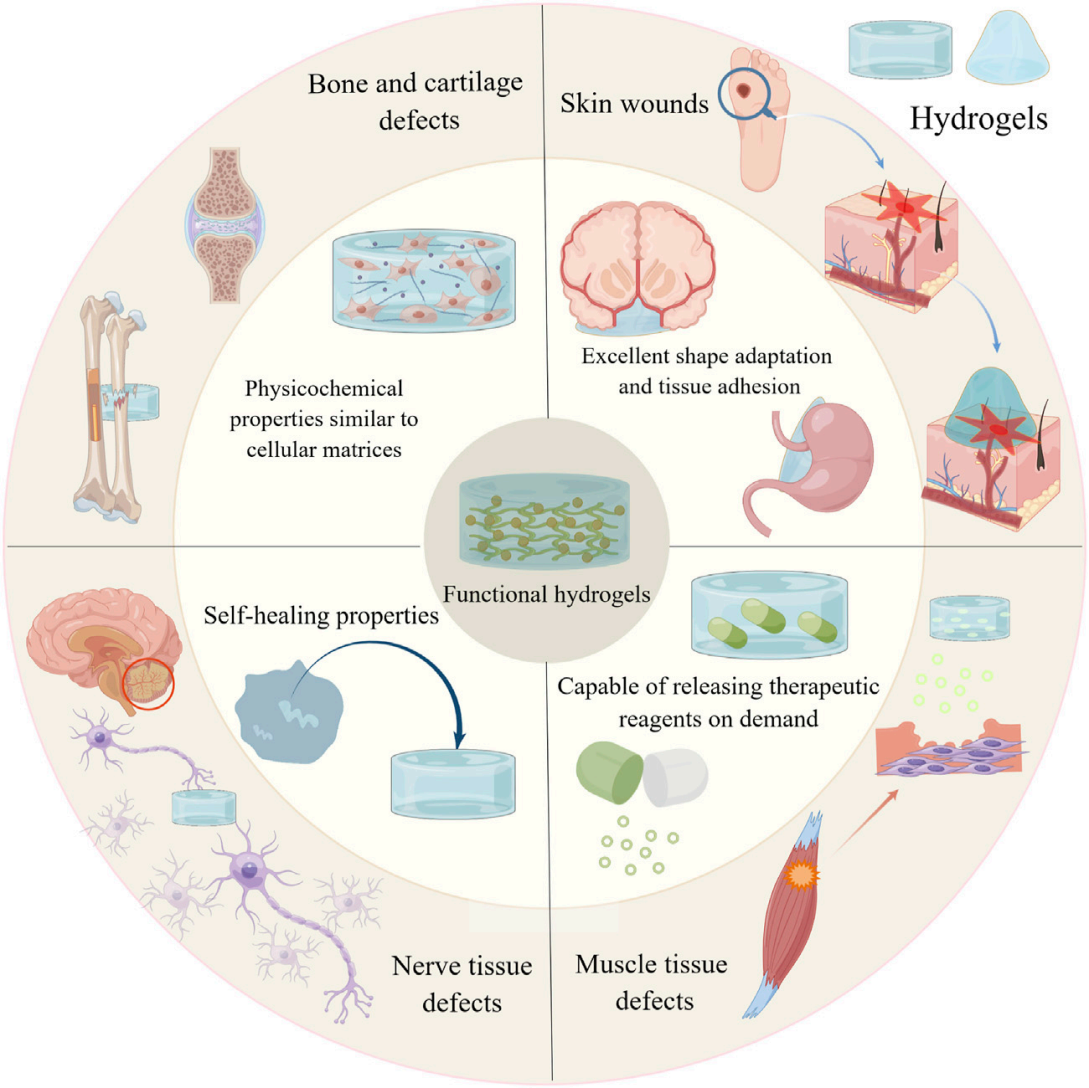
Graphical abstract. By Figdraw (www.figdraw.com).

This paper reviews a variety of functional hydrogels that can facilitate the repair and regeneration of tissue defects. First, we summarise and discuss the four outstanding advantages of hydrogels. Second, we describe the applications and advances of functional hydrogels in the repair and regeneration of defects in bone, cartilage, skin, muscle and nerve tissues. Finally, we discuss the challenges faced by hydrogels in the field of tissue regeneration and provide an outlook on their future trends (Figure 1).

## 2 The outstanding advantages of hydrogels

### 2.1 Physicochemical properties similar to the extracellular matrix of natural tissue

Tissue repair and regeneration cannot be achieved without cell growth, reproduction and differentiation. Cells can sense changes in the surrounding extracellular matrix and perform the corresponding biological responses (Safina and Embree, 2022). The structure, stiffness, degradability, pore size and other physicochemical properties of hydrogels are similar to those of the extracellular matrix of natural tissues, which can provide a good microenvironment for cell proliferation and differentiation. (Xiao et al., 2019; Sun et al., 2021). These physicochemical properties also confer unparalleled biocompatibility to hydrogels. Good biocompatibility is a necessary prerequisite for all biomaterials to be used in biomedical research and clinical applications (Kass and Nguyen, 2022). The structure of a hydrogel is determined by its constituents, synthesis strategy, manufacturing process and gelation method. The diameter of the hydrogel backbone fibres can range from nanometers to microns. The backbone fibre orientation can be adjusted from 0° to 180°. (Hogrebe et al., 2017). Therefore, the structure of hydrogels can be rationally designed according to the structural characteristics of the extracellular matrix in different parts of the body to meet the needs of most tissue repair and regeneration (Volpi et al., 2022). Prochondrocyte differentiation markers appeared upregulated on electrospun chitosan matrices cultured with nanometer diameter fibres, showing higher differentiation potential (Noriega et al., 2012). On electrospun poly (L-lactic) (PLLA) scaffolds with micrometer diameter fibres, RAW 264.7 macrophages were significantly more active and able to secrete more proinflammatory cytokines and chemokines (Saino et al., 2011). The stiffness of the extracellular matrix of different tissues *in vivo*, such as bone, skin, muscle, and nerves, varies widely. When hydrogels are used as tissue extracellular matrix substitutes, cell morphology and cellular behaviors such as proliferation, migration, and differentiation are affected by the stiffness of the hydrogels (Basurto et al., 2022; Yan et al., 2022). It has been reported that mesenchymal stem cells (MSCs) cultured on polyacrylamide hydrogels tend to differentiate into osteoblasts when they are designed to be rigid. In contrast, stem cells cultured on soft polyacrylamide hydrogels have tended to differentiate more into lipogenic cells (Park et al., 2011; Shih et al., 2011). Matrix metalloproteinase-7 (MMP-7) expression was significantly upregulated in human colorectal cancer cells cultured on rigid polyacrylamide hydrogels, and cell proliferation was more active, which is a sign of poor prognosis in colorectal cancer (Nukuda et al., 2015). Moreover, if the stiffness of the hydrogel differs significantly from the natural tissue at the tissue defect, it can lead to stress concentration or even relative motion at the contact interface, which can lead to tissue repair failure. Therefore, the stiffness of the hydrogel should be reasonably designed according to the needs of different sites. Currently, adjusting the specific gravity of nanoparticles in the hydrogel and chemical cross-linking of the hydrogel is the most commonly used method to adjust the hydrogel stiffness (Arno et al., 2020; Du et al., 2020; Lim et al., 2020).

**FIGURE 2:**
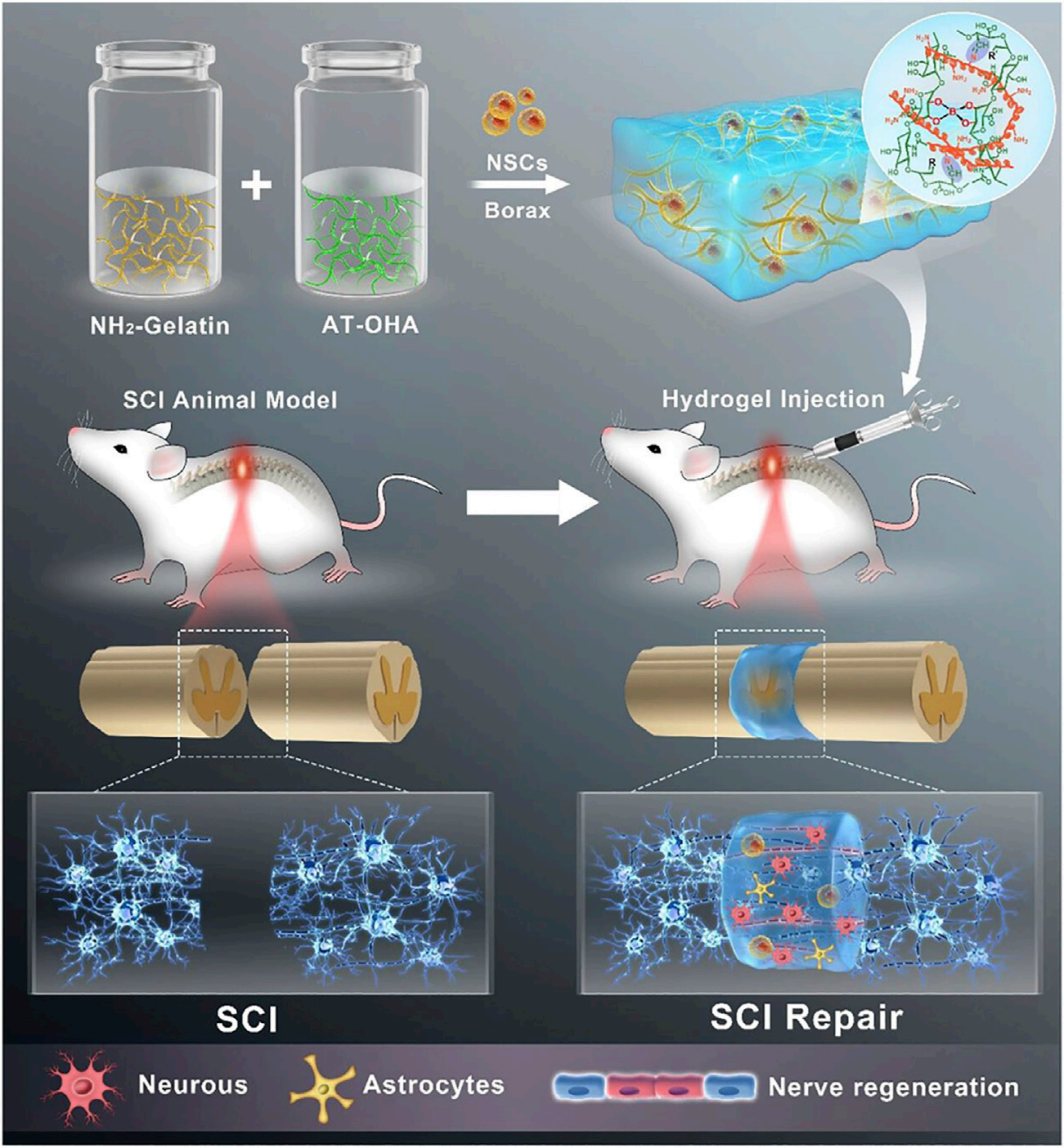
A biodegradable hydrogel based on amino-modified gelatin (NH2-Gelatin) and aniline tetramer grafted oxidised hyaluronic acid (AT-OHA) for spinal cord tissue repair and regeneration. Reproduced with permission (Liu et al., 2023). Copyright 2023, American Chemical Society.

Biodegradability is also an important property of hydrogel materials that can be widely used for tissue repair and regeneration. (Kass and Nguyen, 2022). The metabolism and degradation of the extracellular matrix *in vivo* has been shown to influence cellular behaviors such as cell proliferation, migration, and spreading. When hydrogels have similar degradability to the extracellular matrix, cells in tissue defects can undergo a continuous dynamic remodelling process, which clearly facilitates cell proliferation, differentiation, and collagen and vascular neogenesis (Xu P. et al., 2022; Chang et al., 2022). When hydrogels are loaded with drugs or cells, their controlled degradability allows for a slow and sustained release of drugs and cells (Khan et al., 2022). Liu et al. (2023) reported a biodegradable conductive hydrogel scaffold composed of amino-modified gelatin (NH2-Gelatin) and aniline tetramer grafted oxidised hyaluronic acid (AT-OHA). This hydrogel scaffold has controlled biodegradability and has been shown to promote recovery and regeneration of spinal cord tissue, which has a promising future in the treatment of spinal cord injury and other diseases (Figure 2).

Both hydrogels and extracellular matrices have porous network structures, and their pore sizes are equally important physicochemical properties that affect cell behavior. When hydrogels are used for tissue regeneration, the porous channels will serve as transport channels for cells, drugs, nutrients and metabolites (Zheng et al., 2017; Huang F. et al., 2021). The pore size of hydrogels is highly adjustable. A gelatin methacryloyl hydrogel foam with adjustable pore size was designed by Dehli et al. (2019) This hydrogel foam can be divided into dry foam and liquid foam, while the pore size of dry foam is 15%–20% smaller than that of liquid foam. In addition, the different pore sizes will produce different spatial restrictions on the cells. The different spatial restrictions will activate cells to produce different mechanical transduction signals, thus affecting cellular behaviors such as proliferation, spreading, migration and differentiation (Zeng et al., 2014; Haung et al., 2021). For example, submicron and nanoscale pores can severely restrict human endothelial cell adhesion, proliferation, spreading, and migration, which in turn inhibits angiogenesis at the tissue defect (Li Q. et al., 2021a). Lu et al. (2022) designed methacrylated hyaluronic acid (HAMA) hydrogels with different pore sizes and performed *in vitro* and *in vivo* angiogenesis experiments. It was found that HAMA hydrogels with large pore sizes could promote endothelial cell spreading, proliferation and migration. In contrast, HAMA hydrogels with a medium pore size of 200–250 μm had the best effect on endothelial cell migration and tissue vascularization. In conclusion, the pore size of hydrogels should be designed rationally, and it has a great impact on tissue regeneration.

### 2.2 Excellent shape adaptation and tissue adhesion

Hydrogels are viscoelastic materials with excellent rheological properties (Wang Y. C. et al., 2022). As a result, they have excellent shape adaptation and tissue adhesion properties, for which they can be applied to a variety of irregularly shaped tissue defects and adheres well to the defect to provide continuous and efficient restorative function (Fourmann et al., 2021; Zhou et al., 2022). Tissue defects, whether caused by inflammation, tumors or trauma, are often irregular in shape. Conventional repair requires trimming and reshaping of the defect, which can cause “secondary damage” and further enlarge the defect. In addition, many critical-size defects may be too thin and fragile to support tissue repair and regeneration because the normal tissue becomes thin and fragile or even disappears due to trimming and grinding. Therefore, it is necessary to develop a restorative material that can be used for irregular defects (Huang B. et al., 2022; Gao et al., 2022). Shaowei Zheng et al. (2022) designed a composite hydrogel based on polyvinyl alcohol (PVA), sodium tetraborate (Na2B4O7) and tetraethyl orthosilicate (TEOS). This hydrogel not only has excellent rheological properties and mechanical strength but also accelerates bone reconstruction and healing, which has great potential in the treatment of critical-size segmental bone defects. Jin-A Kim et al. (2021) reported an injectable hydrogel system based on fibrin (Fb) and polyethylene oxide (PEO) for the treatment of meniscal segmental defects. The rheological properties of the hydrogel are highly customizable and can be adjusted by varying the polymer and cross-linker concentrations, polymer molecular weight, polymer reactive group concentration, ambient temperature and pH, preparation method, and gelation method (Piantanida et al., 2019). Richa and Roy Choudhury (2020) then reported a pH-responsive chitosan hydrogel whose stabilising behavior and thixotropy varied with pH. Liang et al. (2019) reported an injectable conductive hydrogel based on hyaluronic acid-graft-dopamine and reduced graphene oxide (rGO). The hydrogel not only has excellent antioxidant activity and self-healing properties but also has tunable rheology for the repair of irregularly shaped skin defects in full layers.

Excellent tissue adhesion is also a major advantage of hydrogels. The hydrogel adheres well to the defective tissue and thus adheres tightly to the wound site for a long period of time, which not only provides physical isolation and a moist environment for the defect but also provides continuous and efficient drug release to promote healing (Yang Y. et al., 2022; Wu et al., 2022). However, tissue defects caused by inflammation, trauma and surgical resection are often accompanied by exudation of body fluids, which requires effective wet adhesion of biomaterials to the defective tissue when used for treatment. Wet adhesion has been a challenge that has limited the application of many biomaterials (Bovone et al., 2021; Wang S. et al., 2022). Inspired by mussel material, Fang-Xue Zhang et al. (2021) developed a highly adhesive hydrogel composed of alginate-dopamine, chondroitin sulfate, and regenerated silk fibroin (AD/CS/RSF). The hydrogel has a shear strength of up to 120 kPa after adhesion to moist tissues, which is promising in the treatment of cartilage defects. Xiaodong Wang et al. (2022) reported a hydrogen-bonded hydrogel (PAAcVI) based on acrylic acid and 1-vinylimidazole. The hydrogel was able to produce tough wet adhesion to tissues by hydrogen bonding. Hong et al. (2019) designed methacrylated gelatin (GelMA) and N-(2-aminoethyl)-4-(4-(hydroxymethyl)-2-methoxy-5-nitrosophenoxy) butanamide linked to glycosaminoglycan hyaluronic acid (HA-NB) in a bionic hydrogel system. The hydrogel has been reported to be extremely wet-adhesive, capable of adhering and sealing arterial and cardiac walls that are acutely bleeding, and has shown promise in the treatment of acute traumatic tissue defects (Table 1).

**TABLE 1:** The outstanding advantages of hydrogels.

Advantages	Characteristics	Representative hydrogels
Physicochemical properties	structure; stiffness; degradability; pore size	electrospun PLLA (Saino et al., 2011); polyacrylamide (Shih et al., 2011); NH_2_-Gelatin/AT-OHA (Liu et al., 2023); HAMA (Lu et al., 2022)
Shape adaptation	rheology	PVA/Na2B4O7/TEOS (Zheng S. et al., 2022); Fb/PEO (Kim J. A. et al., 2021); rGO (Liang et al., 2019)
Tissue adhesion	wet adhesion	AD/CS/RSF (Zhang F. X. et al., 2021); PAAcVI (Wang X. et al., 2022); GelMA/HA-NB (Hong et al., 2019)
Delivery systems	hydrophobic drugs; bioactive reagents; exogenous cells	curcumin/glycosaminoglycan (Zhang et al., 2022); TGF-β3/PLGA/mPA (Lin et al., 2021); hUMSCs/GelMA/Chi-C (Xu et al., 2019)
Controlled release	light; temperature; pH; redox potential; magnetism	HG1-CW (Yang et al., 2020); CS/β-GP/gelatin (Xu et al., 2019); HA/AED (Gao et al., 2019); HA/CMNPs (Daya et al., 2022)
Self-healing properties	physical self-healing; chemically self-healing	Alg-CD/Ad-GO (Soltani et al., 2021); HA-PBA/PVA.

### 2.3 An intelligent delivery system capable of releasing therapeutic reagents on demand

Many domestic and international scholars are working on developing an intelligent delivery system capable of releasing therapeutic reagents on demand for the repair and regeneration of tissue defects (Adepu and Ramakrishna, 2021; Wang X. et al., 2021). Once the therapeutic reagent is released on demand, it can prolong the treatment time, improve the efficiency of the treatment and reduce the side effects caused by the high concentration of the reagent (Li et al., 2023). Therapeutic reagents can be divided into small molecule drugs, bioactive reagents such as growth factors and cytokines, and various exogenous cells. In the repair and regeneration of tissue defects, conventional delivery methods do not produce good results, especially when hydrophobic drugs, bioactive reagents and exogenous cells are delivered. Hydrogels, with their fully interoperable porous structure, are naturally suitable for loading various substances and releasing them on demand at specific locations, offering outstanding advantages in the delivery of therapeutic reagents (Ianchis et al., 2020; Mo et al., 2022).

First, for hydrophobic drugs, their poor solubility under physiological conditions and inability to be used alone lead to their low bioavailability and poor therapeutic efficacy. As a highly absorbent hydrophobic material with a three-dimensional mesh structure, hydrogels can greatly improve the loading rate of hydrophobic drugs and the local concentration of tissue defects (Kass and Nguyen, 2022). Yu et al. (2020) reported a novel hydrogel system based on β-cyclodextrin-modified hyaluronic acid (HA-CD), adamantane-modified 4-arm-PEG (4-arm-PEG-Ad) and hydrophobic dexamethasone. The hydrogel has a hydrophobic cavity and can be loaded with a high concentration of hydrophobic dexamethasone. The hydrogel can modulate the release of dexamethasone by modulating 4-arm-PEG-Ad. Zhang et al. (2022) designed a glycosaminoglycan-based hydrogel delivery system. The hydrogel cleverly encapsulates the hydrophobic drug curcumin using a dynamic supramolecular cross-linked structure and releases it on-demand at the defect, which has promising potential in the repair of chronic skin defects. For the delivery of bioactive reagents, the high susceptibility to deactivation during delivery is an urgent problem to be solved; Rao et al. (2020) reported a nanofibrous hydrogel using aligned chitosan fibre successfully grafted with RGI peptide (Ac-RGIDKRHWNSQGG) and KLT peptide (Ac-KLTWQELYQLKYKGIGG). It was found that the hydrogels successfully prevented the inactivation of these two bioactive peptides during delivery and released them slowly on demand to facilitate the repair of sciatic nerve defects. Lin et al. (2021) reported a methoxy poly (ethylene glycol)-poly (alanine) (mPA) hydrogel capable of loading poly (lactic-co-glycolic acid) (PLGA) microspheres. The growth factor TGF-β3 is encapsulated in PLGA microspheres and can be released continuously and slowly at the site of tissue defects. The delivery of desired exogenous cells to the site of tissue defects for targeted therapy is promising and therefore attracts many scholars. Exogenous cells mainly include stem cells with multidirectional differentiation potential and functional cells such as keratin-forming cells, fibroblasts, and endothelial cells (Chen P. et al., 2021; Kim K. et al., 2021). However, the low cell survival rate, the difficulty of cell transplantation and the inactivation of cells during delivery are serious problems for the development of this field in the traditional delivery process (Benmelouka et al., 2021). Hydrogels have physicochemical properties similar to those of natural extracellular matrices, and their three-dimensional porous meshwork allows for smooth loading of exogenous cells and nutrients required by cells and provides the appropriate biophysical and biochemical cues to maintain cell survival and function. Zhang et al. (2020) designed hybrid RGD-alginate/laponite hydrogel microspheres capable of encapsulating human dental pulp stem cells (hDPSCs) and vascular endothelial growth factor (VEGF) to promote the regeneration of dental pulp tissue. Hongjie Xu et al. (2022) reported a gelatin methacrylate/chitosan-catechol (GelMA/Chi-C) hydrogel capable of encapsulating human umbilical cord mesenchymal stem cells (hUMSCs) to promote diabetic wound repair.

The “intelligence” of the hydrogel is mainly demonstrated by its ability to respond specifically to internal and external environmental stimuli such as light (especially near-infrared light), temperature, pH, redox potential, and magnetism so that its loaded therapeutic substances can be remotely controlled and released on demand (Hao et al., 2021; Mo et al., 2022). The release of this therapeutic substance is time- and space-accurate according to the site and state of the tissue defect, thus maximising the therapeutic efficiency and minimising the side effects (Yang X. et al., 2022). Yang et al. (2020) developed a novel near-infrared light-responsive hydrogel system (HG1-CW) based on dodecyl-modified and Schiff base-linked chitosan, ciprofloxacin, and photothermal agents for promoting healing of infected wounds. Xu et al. (2019) designed an injectable hydrogel system (CS/β-GP/gelatin) based on β-sodium glycerophosphate, chitosan and gelatin. The hydrogel system can specifically respond to changes in temperature stimulation and thus release aspirin and erythropoietin on demand to promote the repair and regeneration of periodontal tissues. Wan et al. (2022) reported a polydopamine-modified hydroxybutyl chitosan hydrogel loaded with aspirin and bone morphogenetic protein-2 (BMP-2) for the repair and regeneration of bone tissue defects. The hydrogel is biresponsive to near-infrared light and temperature and is capable of releasing both drugs on demand. In the early stages of bone defects, the hydrogel provides rapid release of aspirin to improve the early inflammatory response and facilitate the transition to the regenerative phase of the bone defect. A pH/glucose dual response hydrogel system was reported by Liang et al. (2022) The hydrogel specifically releases drugs such as metformin and graphene oxide in response to changes in pH and glucose concentrations in diabetic chronic wounds, thereby greatly accelerating wound healing; Gao et al. (2019) designed a redox-responsive hydrogel based on hyaluronic acid (HA) and aminoethyl disulfide (AED). The hydrogel was particularly sensitive to changes in glutathione (GSH) concentration in the wound and promoted cell growth and tissue regeneration at the wound site. Daya et al. (2022) reported a magnetic hydrogel (HA/CMNPs) based on curcumin, iron oxide magnetic nanoparticles and hyaluronic acid for promoting angiogenesis and tissue regeneration. In conclusion, the hydrogel is a versatile therapeutic biomaterial that provides an effective alternative strategy for the regeneration and repair of tissue defects.

### 2.4 Self-healing properties

**FIGURE 3:**
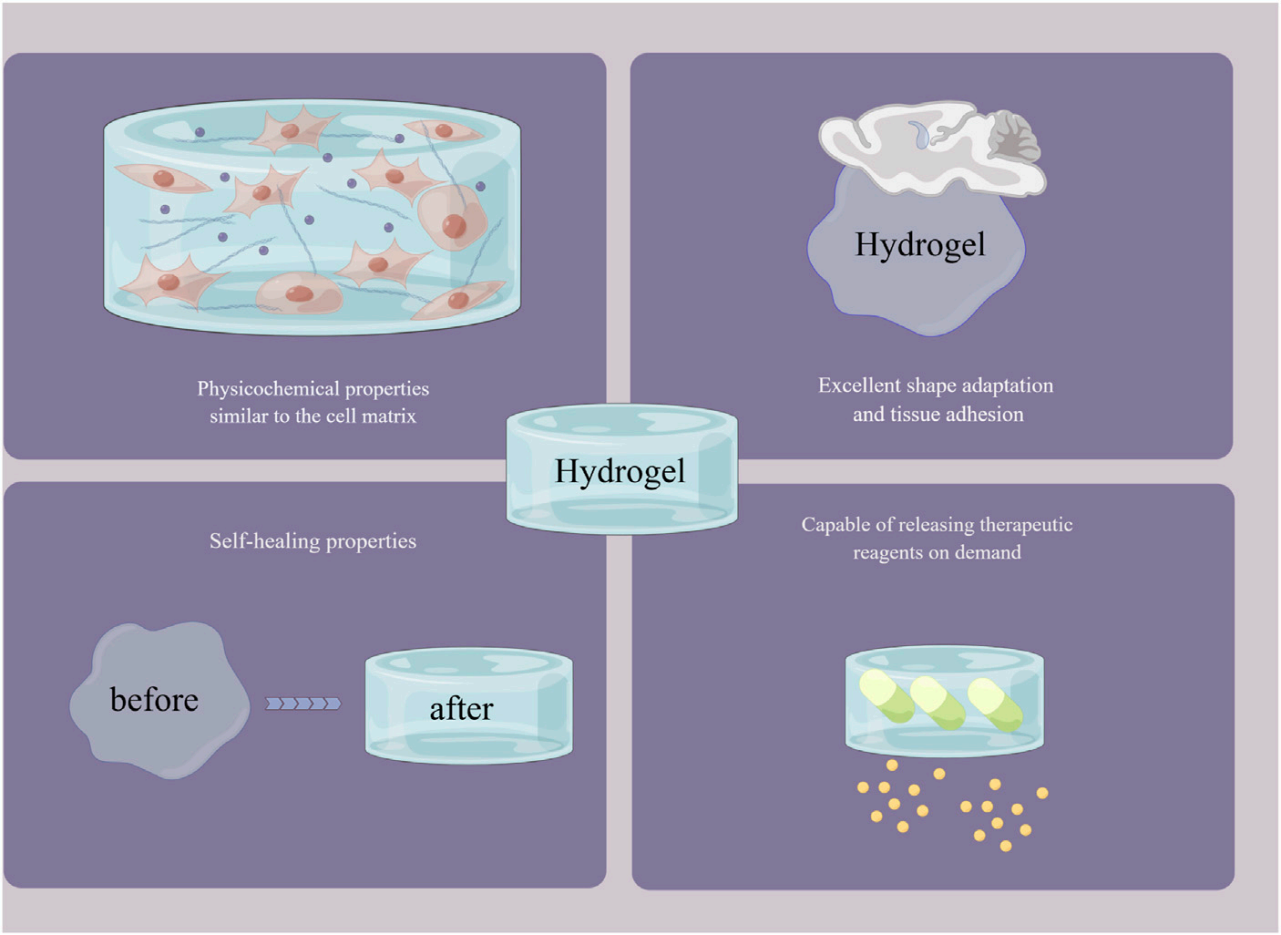
The outstanding advantages of hydrogels. By Figdraw (www.figdraw.com).

Biomaterials are inevitably damaged by external tension, mechanical stress, tissue activity, fatigability, etc., when filling and repairing defects in tissues such as bone, skin and muscle (Zhu et al., 2023). This damage can cause the biomaterial to deform or even rupture and fracture. In addition to further aggravating the defect by “secondary injury” to the tissue, breakage of the biomaterial may also cause external bacterial invasion and lead to wound infection. Therefore, it is necessary to maintain the integrity of the biomaterial during the healing process of the tissue defect (Rumon et al., 2022). Hydrogels are generally polymer networks formed based on dynamic noncovalent interactions or dynamic chemical bonding and have the property of automatically repairing their own structural and functional damage. This self-healing property gives hydrogels an outstanding advantage in tissue repair and regeneration, which has received much attention from scholars both at home and abroad (Quan et al., 2022). Hydrogels with self-healing properties based on dynamic noncovalent interactions such as hydrophobic interactions, host-guest interactions, and hydrogen bonding are considered physical self-healing hydrogels. Meng et al. (2020) then developed a hydrophobic conjugated hydrogel system based on the hydrophobic monomer stearyl methacrylate and amphiphilic regenerated filamentous protein. The hydrogel was formed by combining hydrophobic interactions as sacrificial bonds and exhibited strong mechanical strength, ductility, toughness and good self-healing properties. Soltani et al. (2021) reported a nanohybrid hydrogel formed by host-guest interactions between β-cyclodextrin-modified alginate (host macromere, Alg-CD) and adamantine-modified graphene oxide (guest macromere, Ad-GO), which exhibited excellent rheological properties and self-healing. Zheng Wang et al. (2022) successfully prepared a supramolecular hydrogel based on a monomeric nucleoside molecular gelator (2-amino-2′-fluoro-2′-deoxyadenosine) by constructing a multihydrogen bonding system. The multihydrogen bonding system gives the hydrogel excellent self-healing properties, mechanical strength and shear-thinning injectability, which are promising for the healing of extraction wounds. Hydrogels with self-healing properties based on dynamic chemical bonds such as phenylboronic acid ester bonds, Schiff base structures, and metal ligand coordination are considered chemically self-healing hydrogels. Shi et al. (2020) reported a dynamic self-healing hydrogel system based on phenylboronic acid-modified hyaluronic acid (HA-PBA) and a phenylborate ester bond between poly (vinyl alcohol) (PVA) that exhibited good viscoelasticity, self-healing and injectability. The Schiff base structure is one of the most common structures in self-healing hydrogels (Mo et al., 2021). Runze Li et al. (2022) reported a multifunctional self-healing hydrogel based on a reversible Schiff base reaction between gelatin methacryloyl and oxidised dextran. Xue et al. (2022) successfully prepared a polysaccharide-based hydrogel with excellent self-healing properties by a reversible Schiff base reaction between quaternized chitosan and oxidised hyaluronic acid. Luo et al. (2022) reported a multiligand-derived hydrogel based on sodium alginate, metal ions (Gd^3+^) and diphosphate-functionalized poly (citrate). The multiligand metal ligand pairing gives this hydrogel excellent self-healing properties, injectability and a macroporous structure that facilitates tissue repair (Figure 3).

## 3 Applications of hydrogels for repair and regeneration of tissue defects

### 3.1 Bone and cartilage tissue defects

#### 3.1.1 Articular cartilage defects

Articular cartilage diseases are often degenerative diseases, and their lesions range from articular cartilage to the underlying subchondral bone, which often occur in middle-aged and elderly people. These lesions have gradually become a serious social issue because of their high incidence, the large population involved and serious impacts on quality of life (Wei and Dai, 2021; Qiao et al., 2022). Some hydrogels, which have similar physical and chemical properties to natural cartilage tissue and have properties such as ultrafast gelation and strong adhesion, have shown extraordinary potential in the therapy and repair of articular cartilage diseases. Although the treatment of articular cartilage diseases is very difficult, the continuous development of these hydrogel materials has brought unlimited hope for the treatment of such diseases (Wei et al., 2020; Wang et al., 2023). Physical and chemical properties (such as composition and stiffness) are important factors considered when selecting a stent. Inspired by this, Zhuoxin Chen et al. (2021) developed a heterogeneous double-layer hydrogel scaffold composed mainly of gelatin methacrylate (GelMA) and acryloyl glucosamine (AGA) on the upper layer and vinylphosphonic acid (VPA) on the lower layer. GelMA and AGA were mainly used to simulate the ECM of healthy natural cartilage, and the lower layer of vinylphosphonic acid (VPA) was mainly used to induce chondrogenic differentiation of chondrocytes. Ca^2+^ and alginate were added to the upper and lower layers, respectively, and the upper and lower layers were in close contact with each other through the anchoring of Ca^2+^ and alginate. The combined addition of GelMA and AGA has a strong synergistic effect on the production of type II collagen by chondrocytes, which is 6.7 and 10.8 times stronger than that of the GelMA and AGA groups alone. It is highly promising that high performance hydrogel systems are used for gene carrier delivery to treat articular cartilage defects. Madry et al. (2020) reported a hydrogel system based on PEO-PPO-PEO Poloxamers for the delivery of recombinant adeno-associated virus (rAAV) vectors. This hydrogel system enables *in situ* on-demand release of rAAV vectors and greatly improves the efficiency of treatment of articular cartilage defects.

**FIGURE 4:**
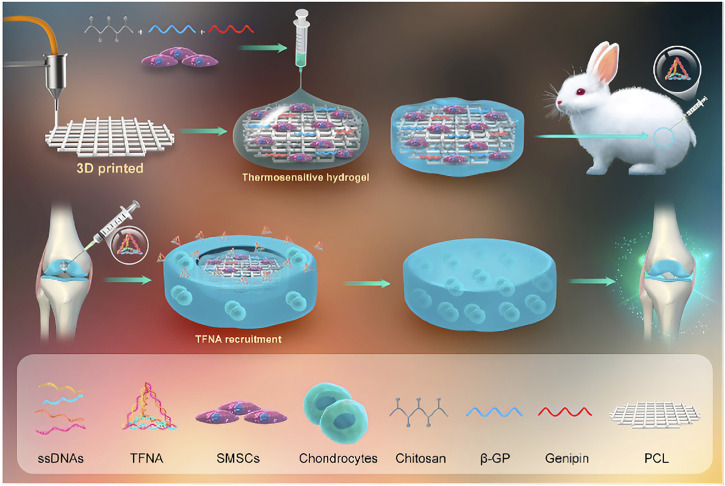
A cartilage damage repair system that uses a chitosan hydrogel/3D-printed poly (ε-caprolactone). (PCL). mixture as a scaffold and contains SMSCs and tetrahedral framework nucleic acid (TFNA). Reproduced with permission (Li P. et al., 2021). Copyright 2021, Elsevier Ltd.

The use of hydrogels loaded with chondrogenesis-related living cells for the treatment of cartilage tissue defects is a hot topic of current research and has great potential to overcome this challenge (Zhang and Khademhosseini, 2017; Mingxin Li et al., 2022). Hua et al. (2021) developed a dual-network (DN) hydrogel scaffold that can be loaded with autologous chondrocytes using hybrid photocrosslinking (HPC) that combines photoinitiated radical polymerisation and photoinduced imine cross-linking. The addition of methacrylate-grafted hyaluronic acid (HA) greatly increases the speed of gelation and keeps the gel time within one second. According to the research, the combination of o-nitrobenzyl (NB) and methacrylate-grafted hyaluronic acid (HA) formed a DN structure, which greatly increased the mechanical strength of the HPC hydrogel to 2 MPa, fully satisfying the requirements for the treatment of articular cartilage disease. The photogenerated aldehyde groups of the HANB adhesive can quickly chemically anchor with the amino groups on the outer layer of the surrounding cartilage tissue, generate strong adhesion, and greatly improve the adhesion of the hydrogel to the cartilage tissue. Finally, the use of a porcine subcutaneous implantation model strongly demonstrated that ACI-loaded dual-network HPC hydrogels can successfully repair articular cartilage defects in the weight-bearing area. Synovial mesenchymal stem cells (SMSCs) are endogenous articular stem cells that have received increasing attention from scholars due to their strong chondrogenic differentiation ability and have been used to treat articular cartilage diseases (To et al., 2019; Matai et al., 2020; Li Q. et al., 2021b). Pinxue Li et al. (2021) reported a cartilage damage repair system that uses a chitosan (CS) hydrogel/3D-printed poly (ε-caprolactone) (PCL) mixture as a scaffold and contains SMSCs and tetrahedral framework nucleic acid (TFNA)**.** The 3D-printed poly (ε-caprolactone) (PCL) mixture has good mechanical strength and provides suitable mechanical support for cartilage damage repair systems. Chitosan is positively charged and can bind to TFNA by electrostatic interactions, so it mainly plays a role in attracting free TFNA in the joint cavity of the body in the system. TFNA is the main functional component of the cartilage repair system. It can improve the microenvironment around SMSCs and promote the proliferation and chondrogenesis of SMSCs, thereby stimulating cartilage regeneration and repairing cartilage defects. Finally, the knee joint experiment using New Zealand white rabbits strongly confirmed that the cartilage repair hydrogel system can effectively promote cartilage regeneration and repair cartilage defects, which is a promising cartilage defect repair strategy (Figure 4).

#### 3.1.2 Bone tissue defects

**FIGURE 5:**
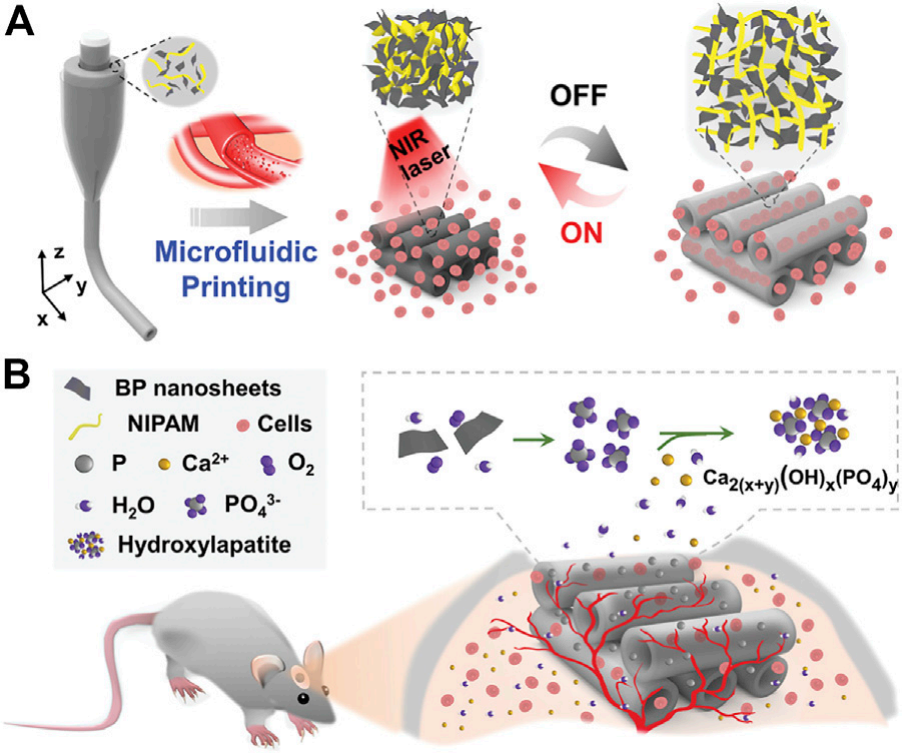
**(A)** Schematic diagram of the reversible contraction and swelling reaction of the dynamic hydrogel system triggered by NIR irradiation, which can promote the penetration of cells into the scaffold channel. **(B)** BP nanomaterial captures free calcium ions to accelerate *in situ* biomineralisation in rat skull defects. Reproduced with permission (Wang X. C. et al., 2022). Copyright 2022, John Wiley & Sons.

Severe orthopedic diseases, trauma, and surgical resection of tumors often cause bone defects that exceed the critical size. Their treatment and repair often bring great challenges and pressure to doctors (Okuchi et al., 2021). Generally, grafts (autograft, allograft, or xenograft) used to repair large bone defects are often ineffective and have very limited indications (Hasani-Sadrabadi et al., 2020). The use of a hydrogel system with strong osteogenic activity and excellent mechanical properties as a bone tissue engineering scaffold offers expansive possibilities in the treatment of an enormous array of bone imperfections (Li J. et al., 2021; Song et al., 2022). Qiu et al. (2020) designed an injectable hydrogel based on periosteal extracellular matrix (PEM). This hydrogel can perform different functions such as regulating inflammation, angiogenesis and osteogenic differentiation at different stages of fracture healing, thus helping to achieve dynamic healing of fracture defects. It is a promising alternative material for bone tissue engineering. Conventional bone tissue engineering scaffolds are mostly solid, so it is difficult to transport nutrients and oxygen to the central area, which obviously results in the inability or delayed growth of bone tissue and blood vessels in the central area. The dynamic channel hydrogel scaffold with reversible contraction and expansion behavior can provide space and channels for the entry of osteoblasts and the generation of blood vessels (Tan J. et al., 2022). Xiaocheng Wang et al. (2022) successfully constructed a dynamic hydrogel system responding to near-infrared light by polymerising a prespinning fluid containing BP, alginate, and N-isopropylacrylamide (NIPAM) from multi-injection glass capillary microfluidic chips through a microfluidic 3D printing strategy. The advantages of this hydrogel system are its sensitive near-infrared light (NIR) response and high thermal conversion efficiency. Reversible shrinkage and expansion will occur when exposed to near-infrared light (NIR). This provides space and channels for the formation of blood vessels and the entry of cells. Additionally, this hydrogel system has strong osteogenic properties because BP nanoparticles can promote osteoblast proliferation and osteogenic differentiation by trapping surrounding calcium ions and accelerating *in situ* biomineralisation (Figure 5).

For large bone defects of critical size, bone tissue engineering scaffold materials used to repair the defect often require extremely strong mechanical properties and osteoconductivity. The mechanical strength and osteoconductivity of many hydrogel materials that have been developed to date still need to be further enhanced because they are often characterised by isotropic network structures (Sano et al., 2018; Fang et al., 2019). After discovering that natural bones and wood have a unique multiscale layered anisotropic structure, Xiaofei Wang et al. (2021) decided to combine delignified natural pine wood with a sodium alginate (SA) hydrogel with good biocompatibility to construct a biomimetic hydrogel composite with a high degree of anisotropy. Due to the advantage of well-arranged cellulose fibrils in delignified natural pine wood, the composite has excellent mechanical properties (tensile strength = 67.8 MPa, elastic modulus = 670 MPa), which is nearly 3 orders of magnitude higher than that of pure sodium alginate (SA) hydrogel and surpasses almost all conventional hydrogel materials. Severe bone hemorrhage is a common complication of large bone defects caused by trauma and tumor resection. A large amount of bone bleeding often hinders the operation and seriously affects the repair of bone defects, which brings great challenges to medical treatment. Traditional hemostatic materials often rely on compression to stop bleeding, which is not conducive to the formation of new bone in bone defects. They also do not have excellent osteogenic activity and adhesion, so they are obviously not suitable for repairing bone defects as bone tissue engineering scaffolds (Huang T. et al., 2022; Bian et al., 2022; Tan X. et al., 2022). To solve this problem, Weijuan Huang et al. (2021) successfully developed a composite hydrogel material that has good adhesiveness and can effectively stop bleeding and promote bone regeneration by a dynamic Schiff base reaction between the aldehyde groups of aldehyde-modified cellulose nanocrystals (DACNC) and the amine groups of catechol-coupled chitosan (CHI-C). The composite hydrogel material can be injected into the bone defect area with heavy bleeding and can react quickly within 2 min. It can strongly adhere to the bleeding site and completely fill the irregularly shaped bone defect, successfully stopping bleeding and repairing the defect.

Osteoporosis is a serious type of bone disease that is difficult to completely heal and exhibits a high incidence and many complications. It is often due to the imbalance between bone resorption and bone formation in the process of bone metabolism. Osteoporosis patients, especially elderly patients, often suffer from fractures and bone defects due to poor bone regeneration and bone loss, which seriously affect their quality of life (Arceo-Mendoza and Camacho, 2021; Reid and Billington, 2022). The emergence of injectable hydrogel systems with good biocompatibility, suitable mechanical strength, excellent osteogenic activity, and easy use has opened a new direction for the treatment of bone defects in patients with osteoporosis, whose bone defects are more difficult to heal than normal bone defects. Kuang et al. (2021) reported an injectable multifunctional hydrogel scaffold whose injection matrix is composed of *in situ* generated calcium phosphate nanoparticle (ICPN)-coordinated poly (dimethylaminoethyl methacrylate-co-2-hydroxyethyl methacrylate) (DHCP) hydrogel glue. This hydrogel was loaded with near-infrared (NIR) light-modulated thermosensitive polymer microspheres (PIP MSs), which can promote the release of parathyroid hormone (PTH) and stimulate the formation of *in situ* pores on demand to achieve bone regeneration in an ovariectomy (OVX) model (osteoporosis model). Studies have shown that zinc ions and magnesium ions can promote the healing of cancellous bone defects by stimulating the proliferation of osteoblasts and increasing the osteogenic activity of osteoblasts. Xin Chen et al. (2021) constructed a protein cross-linking hydrogel system with a T4 lysozyme mutant (T4M) as the framework and an integrin receptor-binding Arg-Gly-Asp (RGD) sequence connected to the C-terminus of T4M. The protein cross-linked hydrogel system has abundant free amine groups on the outer layer of T4M, which can load a large amount of Mg^2+^ and Zn^2+^ and release Mg^2+^ and Zn^2+^ continuously in order. Zhenyu Zhao et al. (2021) developed a bisphosphonate-functionalized injectable hydrogel microsphere with methacrylonitrile acylation gelatin as the injection matrix and chelated Mg^2+^ (GelMA-BP-Mg). The grafting of bisphosphonate (BP) and chelating Mg^2+^ on the GelMA scaffold were performed through a Schiff base reaction and the coordination reaction of metal ion ligands, respectively. Through related characterisation tests, it was found that GelMA-BP-Mg microspheres with minimally invasive injection and bone targeting capabilities have a strong ability to capture Mg^2+^ (the capture ratio is 0.6%) and enable sustained and effective release of Mg^2+^ in the environment (the effective release time is 18 days).

### 3.2 Skin wounds

Multiple types of wounds often leave many experienced physicians at a loss. However, the emergence of various composite hydrogels brings hope to treatment. These composite hydrogel platforms have many advantages when applied to wound dressings compared to traditional methods. Next, this paper mainly introduces the use of hydrogels to promote the healing of infected wounds, burn wounds and wounds in diabetic patients.

#### 3.2.1 Infected wounds

**FIGURE 6:**
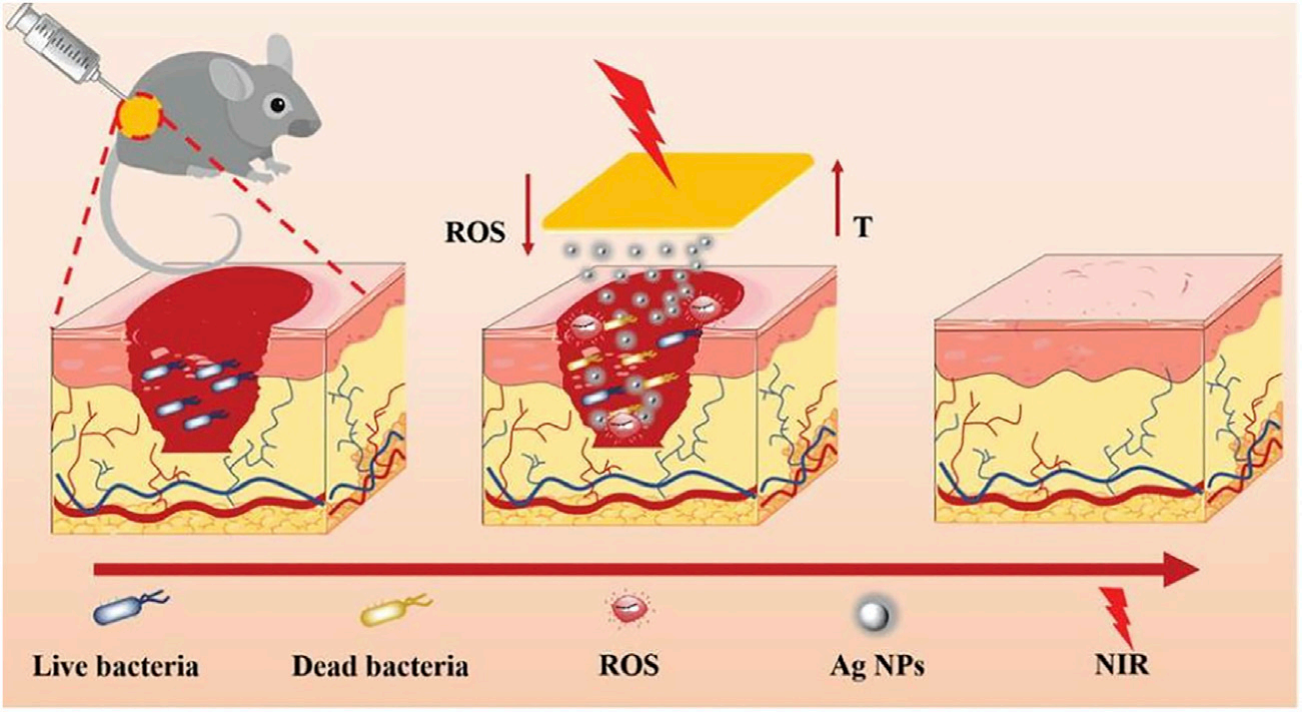
Schematic diagram of Gel-DA/GG@Ag NPs used as wound dressings for wound healing. Reproduced with permission (Zhang H. et al., 2021). Copyright 2021, John Wiley & Sons.

In the recovery process of wounds, bacterial infection is prone to occur and is often accompanied by long-lasting inflammation. It often causes the wound to become chronic and nonhealing and may even further cause serious complications (such as sepsis), which seriously affect the health of the patient (Huang et al., 2020). In this respect, it is extremely important to set up an injury dressing that can be effectively antibacterial and eliminate inflammation. This has important value for accelerating wound healing and avoiding many serious complications (Xiang et al., 2019). With the emergence of multidrug-resistant bacteria, although antibiotics are currently the most common method of treating infected wounds, other effective strategies still must be sought (Xu L. et al., 2022). Multifunctional hydrogel wound dressings can not only exert antibacterial, adhesion, hemostasis, anti-inflammatory, antioxidant and other functions but also achieve physical isolation and create a moist environment, bringing great hope for the treatment of infected wounds (Xiang et al., 2019). Hui Zhang et al., 2021) novatively developed a multifunctional composite hydrogel material (Gel-DA/GG@Ag NPs) by adding guar gum (GG) and boric acid to a mixture formed by the reaction of Ag nanoparticles (Ag NPs) and dopamine-modified gelatin (Gel-DA). The composite hydrogel can effectively release silver ions and has an efficient near-infrared light (NIR) photothermal effect. When exposed to 808 nm near-infrared (NIR) lasers, silver ions and photothermal effects work synergistically to quickly kill bacteria and promote wound healing. In conclusion, Gel-DA/GG@Ag NPs will become a hotspot in the development of multifunctional wound dressings to repair chronic nonhealing infected wounds and other wounds overexpressing reactive oxygen species. Yingnan Liu et al. (2020) successfully developed a composite hydrogel platform (AM NS-CS) that can effectively sterilise through high-efficiency photothermal effects by combining antimony nanosheets (AM NSs) with chitosan (CS). It is worth mentioning that this is the principal article describing AM NSs as a photothermal antibacterial agent applied to the treatment of bacterially infected wounds, which is extremely innovative. The AM NS-CS platform has extremely powerful antibacterial ability and can effectively kill Gram-positive bacteria (*Staphylococcus aureus*, 100%) and Gram-negative bacteria (*E. coli*, 97.1%) (Figure 6).

Wounds infected by methicillin-resistant *S. aureus* (MRSA) often cannot be cured with many commonly used antibiotics. MRSA often causes wounds to dehisce and discharge pus, leading to long-term nonhealing and making patients miserable (Lee et al., 2018; Turner et al., 2019). To solve this problem, Wenshuai Liu et al. (2020) used dihydroxy- and oxadiazole-group-decorated quaternary ammonium salts (QAS) and poly (ε-caprolactone)-poly (ethylene glycol)-poly (ε-caprolactone) (PCEC) copolymer as raw materials to synthesise a nanocomposite hydrogel material that can effectively heal wounds infected by MRSA. The composite hydrogel quickly and effectively kills bacteria such as MRSA, *E. coli* and vancomycin-resistant staphylococci and rapidly heals infected full-thickness skin wounds within 12 days Zhao et al. (2020) successfully constructed a dual-network (DN) hydrogel material that can efficiently act as a photothermal antibacterial agent and accelerate wound healing. It comprises catechol--Fe^3+^ coordination cross-linked poly (glycerol sebacate)-co-poly (ethylene glycol)-g-catechol and quadruple hydrogen bonding cross-linked ureido-pyrimidinone modified gelatin. The composite hydrogel material can quickly and effectively kill multidrug-resistant bacteria, such as methicillin-resistant *S. aureus* (MRSA), under near-infrared light (NIR) irradiation and has excellent bactericidal ability and NIR responsiveness. More importantly, the double-network hydrogel can effectively reduce wound oxidation, control inflammation, accelerate the formation of granulation tissue and blood vessels, and significantly promote the closure and healing of infected wounds.

#### 3.2.2 Burn wounds

The World Health Organisation has reported that approximately 11 million people suffer burns every year (Forjuoh, 2006). Burns are inevitably accompanied by infection, pain and dehydration, which can cause disfigurement, disability and even death. Burn wounds are accompanied by inflammation, loss of body fluids, tissue damage and loss of tissue barrier function, making them very difficult to heal (Church et al., 2006). In addition, a large amount of exudate will appear in a burn wound, so the dressing needs to be changed many times, which not only consumes considerable manpower and material resources but also inevitably damages the tissue and delays healing (Loo et al., 2014). Cook et al. (2021) developed a new type of soluble composite hydrogel dressing produced by the *in situ* reaction of polyethyleneimine (PEI) and NHS-activated polyethylene glycol (PEG) containing internal thioester bonds. This dissolvable hydrogel with good adhesion properties is an ideal burn wound dressing material. The hydrogel can not only physically isolate the wound from the external environment but also efficiently absorb wound exudate and exhibits mechanical properties and elasticity of the same order of magnitude as that of epithelial tissue. Tang et al. (2021) used catechol-modified chitosan (CHI-C), oxidised dextran (ODex) and MgO as raw materials to construct a composite hydrogel doped with metal oxides (CCOD-MgO). Due to the doping of metal oxides, CCOD-MgO not only produces excellent antibacterial activity but also greatly shortens the gel time and improves the mechanical strength. In addition, the incorporation of MgO can also reduce the swelling rate of the hydrogel, thereby reducing the pressure on the burn tissue. The *in vivo* full-thickness skin defect and deep skin burn model proved that CCOD-MgO can effectively promote skin wound healing and tissue regeneration and has an excellent therapeutic effect on severe burn wound infection.

For the unavoidable moving parts of the human body (such as the groin, joints, etc.), the wound dressing will repeatedly rub and collide with the burn wound to damage the wound and cause the wound to last for a longer time (Huang et al., 2018). Thus, the shape adaptability, adhesiveness, abrasion resistance and healing properties of wound dressings face higher requirements. Therefore, Yuan et al. (2021) developed a new type of double cross-linked AG-OD-Fe(III) hydrogel for such burn wounds. AG-OD-Fe(III) hydrogels were prepared by Schiff base cross-linking between catechol-modified oxidised hyaluronic acid (OD) and aminated gelatin (AG) under different–CHO/-NH_2_ ratios and coordination cross-linking between OD and Fe^3+^. AG-OD-Fe(III) hydrogels have excellent shape adaptability (97.1% ± 1.3% recovery rate) and adhesion performance of up to 19.3 kPa. In addition, the hydrogel can quickly undergo a gel reaction in approximately 50 s–54 s and has close to 100% antibacterial activity and excellent hemostatic properties. It is worth noting that AG-OD-Fe(III) hydrogels have excellent wear resistance, which is extremely important for dynamic burn wound healing. The mechanical property tests revealed that AG-OD-Fe(III) hydrogels can withstand compressive stresses up to 611.7 kPa and have excellent mechanical stability, recovery and fatigue resistance, fully meeting the requirements of dynamic burn wound dressings for abrasion resistance. *In vivo* experiments showed that after using the hydrogel dressing for only 13 days, the secondary burn wounds healed, and the skin and blood vessels were reconstructed, which proves that it is an ideal burn dressing. In short, the continuous development of various composite hydrogel dressings with high adhesion, high mechanical strength, low swelling rate, high self-healing properties and excellent shape adaptability brings hope to the healing of many severe burn wounds.

#### 3.2.3 Diabetic wounds

With the continuous improvement of living conditions, the prevalence of diabetes continues to rise. According to statistics, in 2014, there were approximately 400 million diabetic patients, and the quantity of patients is projected to reach 600 million by 2035 (Guariguata et al., 2014). Diabetes patients endure systemic effects such as hyperglycemia, long-term inflammation, impaired angiogenesis, tissue hypoxia and excessive production of reactive oxygen species, which inhibit the proliferation of fibroblasts, keratinocytes and endothelial cells. Therefore, once a diabetic patient is injured, angiogenesis is impaired, and the patient is also prone to uncontrolled infection and inflammation, leading to unhealed or even worsened wounds (Gurtner et al., 2008; Mao et al., 2017). The control and healing of diabetic wounds is still an urgent clinical challenge.

Polarising macrophages from the M1 phenotype to the M2 phenotype through immunomodulation, thereby improving the inflammatory state and promoting angiogenesis, is an effective method to promote the healing of diabetic wounds (den Dekker et al., 2019). Thus, Tu et al. (2021) developed a hydrogel scaffold (GDFE) formed by the dynamic cross-linking reaction of peptides, polydopamine and graphene oxide to efficiently regulate the polarisation of macrophages to the M2 phenotype. GDFE not only shows strong antibacterial, antioxidant and anti-inflammatory abilities but also significantly accelerates the regeneration of blood vessels and promotes the healing of diabetic wounds. It is an ideal diabetic wound dressing. Similarly, to promote angiogenesis and phenotypic transformation of macrophages, Kai Wang et al. (2022) successfully constructed a chitosan-graft-aniline tetramer composite hydrogel loaded with exosomes (CS-AT-Exo). It was found through experiments that exosomes and hydrogel can have a synergistic effect, the efficiency of CS-AT-Exo hydrogel macrophage polarisation to the M2 phenotype was further improved, and the healing efficiency of diabetic wounds was also further improved.

Lu et al. (2021) innovatively developed a heparin-poloxamer hydrogel material that can be loaded with live lactococci (LHP)**.** In addition to promoting the abovementioned phenotypic transition of macrophages, the LHP hydrogel can also promote an increase in vascular endothelial growth factor (VEGF) secretion and directly promote the formation of blood vessels. More importantly, through these two mechanisms, the LHP hydrogel can promote angiogenesis and precise spatiotemporal control of inflammation to drive the wound microenvironment to heal quickly. Therefore, this material is also another potential scaffold for diabetic wounds. Obviously, a reasonable design of the composite hydrogel dressing can efficiently realise the phenotypic transition of macrophages from M1 to M2 and promote the healing of diabetic wounds. This will become an important perspective for treating diabetic wounds that are difficult to heal.

As mentioned earlier, chronic hypoxia and excessive production of reactive oxygen species in tissues represent another major reason why diabetic wounds are not easy to heal. Chronic hypoxia can also severely inhibit angiogenesis, and it can be aggravated by the accumulation of inflammatory cells with high oxygen consumption (Tandara and Mustoe, 2004; Schreml et al., 2010). Therefore, continuous oxygenation is obviously another effective way to promote diabetic wound healing. However, conventional wound dressings and hyperbaric oxygen therapy obviously cannot achieve continuous oxygenation, and composite hydrogel materials can solve this problem. Guan et al. (2021) successfully developed an injectable hydrogel capable of loading oxygen-releasing microspheres (OMs) as a continuous oxygenation delivery system. Most conventional oxygen release systems usually release toxic H_2_O_2_. In contrast, ORM can directly release a large amount of oxygen and continue to release oxygen for at least 2 weeks. In addition, the hydrogel can absorb elevated ROS in tissues, reducing its damage to tissues and cells. The hydrogel, which can continuously release oxygen, provides a new idea for the healing of diabetic wounds and offers important clinical value.

### 3.3 Muscle tissue defects

Muscle tissue, which accounts for more than 50% of body weight, is the only contractile tissue in the body and is critical in force production, body movement, postural support, and maintenance of visceral function. Muscle tissue is a soft tissue that functions and is therefore very susceptible to injury, resulting in impairment of body movement and organ function. Although muscle tissue is self-repairing from minor injuries, self-repair will fail once there is a serious injury with a loss of mass greater than 20% (Nuge et al., 2021; Jeong et al., 2022). Therefore, there is an urgent need for an excellent biomaterial that can serve as a scaffold in muscle tissue engineering to promote its regeneration and repair. Many biomaterials used to fill and repair muscle tissue defects break and fracture due to external tension, mechanical stress, tissue activity, fatigability, and other factors. Hydrogels with high mechanical strength, excellent tissue adhesion and self-repair properties have outstanding advantages in the repair of muscle tissue defects (Zhao J. et al., 2021; Peper et al., 2021).

Skeletal muscle consists primarily of uniaxially ordered myotubes and widely distributed capillaries, which account for approximately 40% of body weight and are the largest tissue type in the human body. Skeletal muscle plays a crucial role in skeletal support, stability, metabolism and movement (Quigley et al., 2021; Jo et al., 2022). The function of skeletal muscle is closely related to its uniaxial orientation and dense arrangement of muscle fibres. Therefore, once the skeletal muscle is damaged to a large extent and the muscle fibres become disorganised or broken, the function of the skeletal muscle will be severely impaired (Sonaye et al., 2022; Pinton et al., 2023). The development of hydrogel materials as bionic functional skeletal muscle structures for filling or replacing damaged tissues is a promising therapeutic strategy in skeletal muscle diseases (Philips et al., 2022). Hydrogels have physicochemical properties similar to the extracellular matrix of natural muscle tissue, for which they are able to accommodate myoblasts or MSCs and provide appropriate biochemical cues to ensure proper differentiation and alignment of the cells. Shan et al. (2021) reported a novel ROS-scavenging hydrogel loaded with mesenchymal stem cells (MSCs). The hydrogel not only improved the viability of MSCs and promoted their differentiation into myoblasts but also promoted the polarisation of M2 macrophages to suppress the local inflammatory response, which significantly promoted the reduction of further skeletal muscle damage and accelerated skeletal muscle regeneration. Alheib et al. (2022) designed a laminin-biofunctionalized gellan gum hydrogel capable of loading myogenic cells for the treatment of moderate muscle injuries that exceed the ability of muscle tissue to heal itself. Hydrogels have unique bionic properties in that their three-dimensional skeletal fibres can mimic the uniaxially oriented muscle fibres of natural skeletal muscle by adjusting the synthesis strategy and manufacturing process; Lee et al. (2022) successfully fabricated artificial functional skeletal muscle using photocrosslinked hydrogels with the help of Microvalve-Assisted Coaxial 3D Bioprinting for tissue reconstruction after muscle defects. Hydrogels with tissue adhesion and self-healing properties can withstand external tension, mechanical stress and fatigue during the repair of skeletal muscle; Carleton et al. (2021) reported a multifunctional hydrogel (MAA-collagen) based on methacrylic acid and collagen for the treatment of volumetric muscle loss. Quint et al. (2022) designed a multifunctional gelatin methacrylate-based hydrogel for the treatment of extreme muscle injuries. In addition, the development of an anisotropic hydrogel scaffold with precisely topographic cues for the treatment of skeletal muscle defects is very prospective. Ling Wang et al. (2022) designed a remote magnetic nanofiber/hydrogel multiscale scaffold for the treatment of volumetric muscle loss. The scaffold can facilitate functional recovery of damaged skeletal muscle by providing cues to cell alignment through remote magnetic fields.

**FIGURE 7:**
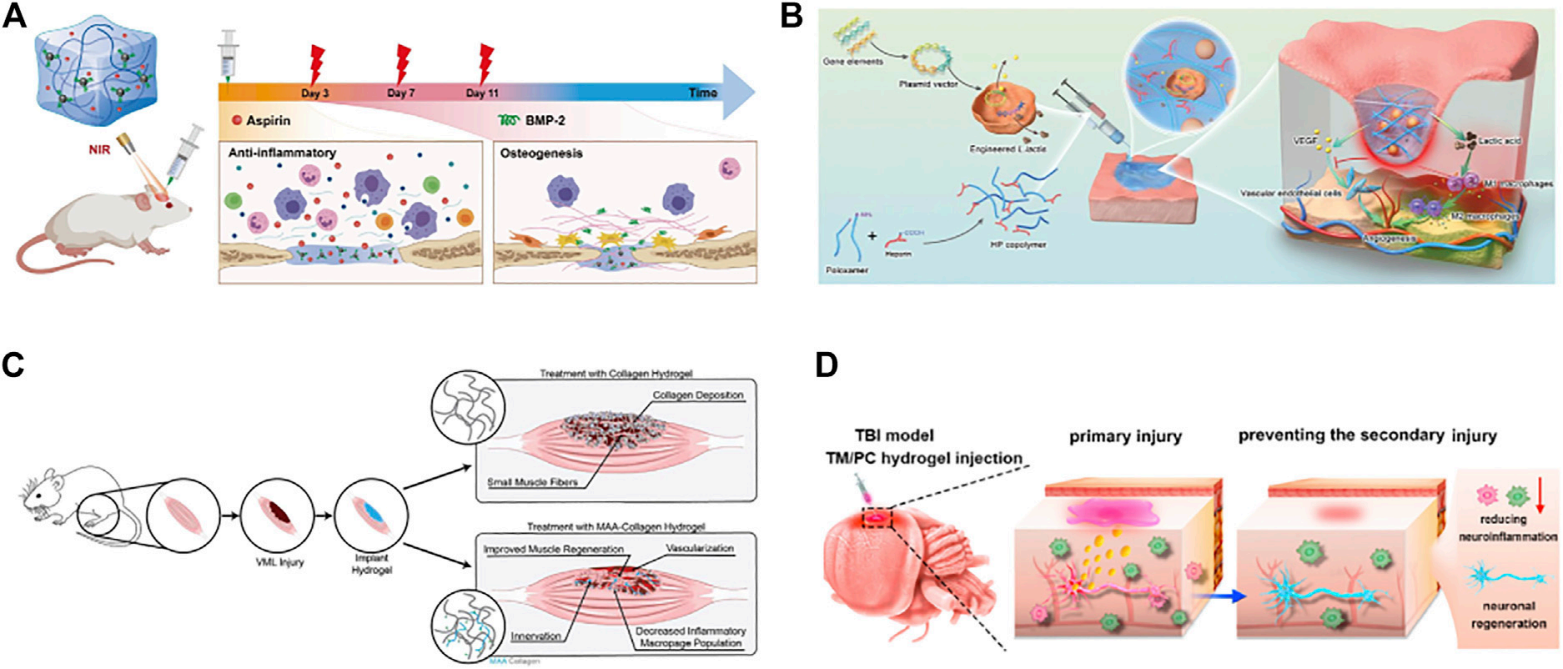
Functional hydrogels can be used for repair of bone, skin, muscle and nerve tissue defects. **(A)** polydopamine-modified hydroxybutyl chitosan hydrogel loaded with aspirin and bone morphogenetic protein-2 (BMP-2) for the repair and regeneration of bone tissue defects. Reproduced with permission (Wan et al., 2022). Copyright 2022, Elsevier Ltd. **(B)** Schematic diagram of the mechanism of action of the LHP hydrogel in promoting diabetic wound healing. Reproduced with permission (Lu et al., 2021). Copyright 2021, John Wiley & Sons. **(C)** Methacrylic acid-based hydrogels enhance skeletal muscle regeneration after volumetric muscle loss in mice. Reproduced with permission (Carleton et al., 2021). Copyright 2021, Elsevier Ltd. **(D)** An injectable hydrogel based on curcumin, poly (propylene sulfide). 120 and matrix metalloproteinase-responsive triglycerol monostearate for repair after brain injury. Reproduced with permission (Qian et al., 2021). Copyright 2021, Elsevier Ltd.

Myocardial infarction and heart failure are currently the leading causes of high mortality in cardiovascular disease. In myocardial infarction, significant apoptosis and loss of myocardial cells occur, and the damaged myocardium undergoes pathological remodelling of tissue fibrosis due to insufficient self-repair capacity (Jenča et al., 2021; Zou et al., 2021). Heart transplantation is currently the preferred treatment strategy for severe myocardial infarction and heart failure but can be limited by the complexity of the procedure, immune rejection, and lack of a donor organ (Kawaguchi et al., 2021). Currently, the use of biomimetic functional materials for myocardial repair and regeneration after injury is a promising therapeutic strategy. However, most materials do not adequately mimic the composition and structure of the natural myocardial tissue extracellular matrix, nor do they provide effective microenvironmental cues for stem cell growth and differentiation (Saludas et al., 2017). Hydrogels are three-dimensional (3D) hydrophilic polymer networks with physicochemical properties similar to those of the natural extracellular matrix (ECM) of myocardial tissue. Moreover, the mechanical properties of hydrogels are similar to those of cardiac tissue, providing suitable mechanical support in filling myocardial tissue defects and transmitting suitable mechanical signals to attenuate unfavorable pathological remodelling of myocardial tissue (Peña et al., 2018; Liao et al., 2020). Li et al. (2020) then designed a double cross-linked injectable hydrogel based on hyaluronic acid. The hydrogel was delivered to the myocardium by percutaneous injection and mechanically bound the infarcted myocardial tissue to limit its pathological remodelling and expansion. Haiting Chen et al. (2022) reported a gelatin methacrylate microneedle (MN) patch loaded with galunisertib for the treatment of myocardial infarction and promotion of cardiac repair. The hydrogel not only provided mechanical support to the fragile ventricular wall but also sustained the release of galunisertib, a transforming growth factor-β (TGF-β)-specific inhibitor, which effectively inhibited uncontrolled and excessive fibrosis of the damaged myocardium. The hydrogel acts as an intelligent delivery vehicle for the injection of therapeutic components (drugs and cells) directly into the myocardium and continuous on-demand release (Zhu et al., 2021). Jiang et al. (2022) developed a pH- and temperature-responsive injectable hydrogel (poly (chitosan-co-citric acid-co-N-isopropyl acrylamide), P(CS-CA-NIPAM)) loaded with oncostatin M. The hydrogel reacted with the acidic microenvironment of myocardial tissue during myocardial infarction to release oncostatin M on demand, which accelerated myocardial cell proliferation and inhibited fibrosis. The hydrogel reacts with the acidic microenvironment of myocardial tissue during myocardial infarction to release oncostatin M on demand, which accelerates cardiomyocyte proliferation and inhibits myocardial fibrosis. Burgess et al. (2021) reported a functionalized peptide hydrogel scaffold capable of delivering cardiac progenitor cells for repair after myocardial injury; Lyu et al. (2020) prepared an injectable hyaluronic acid-based hydrogel loaded with human mesenchymal stem cell aggregates. Injection of this hydrogel into infarcted myocardial tissue effectively improved the microenvironment of myocardial tissue, reduced the expression of inflammatory cytokines and increased the secretion of angiogenic factors (Figure 7).

### 3.4 Nerve tissue defects

Currently, the incidence of neurological deficits such as stroke, Parkinson’s disease, Alzheimer’s disease, Huntington’s disease, and spinal cord injury is increasing dramatically (Bordoni et al., 2020). These diseases may cause speech difficulties, cognitive impairment, memory loss, dementia, depression, disability and even death, causing great suffering to patients and their families. Moreover, neurological disorders are very difficult to treat and have a very poor prognosis, making them one of the most challenging clinical problems in the world (Detante et al., 2014; Tian et al., 2021). Autologous nerve grafting is currently the accepted gold standard for the treatment of nerve injury. However, surgical difficulties, limited availability of autologous nerves and the potential for neurological dysfunction in the donor region have limited the application of autologous nerve grafts (Sun et al., 2022). The development of bioactive materials that induce neural tissue repair and regeneration for the treatment of neurological deficiency diseases is very promising and holds a good chance for overcoming this challenge.

As an intelligent delivery system capable of releasing therapeutic substances such as neuroprotective agents, nutrients, stem cells and growth factors on demand, hydrogels have outstanding advantages in the repair and treatment of neurological tissue defects (Lv et al., 2022). Currently, many drug carriers have difficulty in allowing therapeutic substances to cross the blood‒brain barrier and reach the central nervous tissue. Although some drug carriers allow the therapeutic substance to cross the blood‒brain barrier and be released at high initial concentrations, these newly released therapeutic substances are rapidly absorbed by the cerebrospinal fluid, resulting in a dramatic decrease in local concentration. In contrast, hydrogels loaded with therapeutic substances can be injected in liquid form into the neurological defects of the brain and then gelated *in situ*, which greatly reduces the difficulty of crossing the blood‒brain barrier (Pakulska et al., 2012; Flégeau et al., 2017). Moreover, the hydrogel serves as a stimulus-responsive biocompatible carrier system that can continuously release therapeutic substances on demand and maintain their long-term function at the brain site (Li D. et al., 2022; Grimaudo et al., 2022). Luyu Wang et al. (2021) reported an injectable hyaluronic acid *in situ* hydrogel loaded with bone mesenchymal stem cells and nerve growth factor for the repair treatment of traumatic brain injury. Pradhan et al. (2019) developed an injectable hydrogel based on acetylcholine-functionalized graphene oxide and polyacrylic acid. The hydrogel was injected into the brain to rapidly fill tissue defects, promote neuronal growth and stabilise microtubule networks, which is very promising in the regeneration of neural tissue. Qian et al. (2021) designed an injectable hydrogel (Cur-TM/PC) based on curcumin, poly (propylene sulfide) 120 and matrix metalloproteinase-responsive triglycerol monostearate for repair after brain injury. The hydrogel responds to the tissue microenvironment after brain injury and releases curcumin consistently and efficiently to remove uncontrolled reactive oxygen species from brain tissue and promote neuronal regeneration and recovery.

Hydrogels have adjustable mechanical strength and good biodegradability. Therefore, during the initial phase of nerve tissue repair, the hydrogel can perfectly fill the nerve tissue defect and provide mechanical support for the growth of new tissue. As the repair proceeds, the hydrogel gradually degrades to provide space for the new nerve tissue (Niemczyk et al., 2018). Ghuman et al. (2018) reported a biodegradable extracellular matrix hydrogel based on porcine-derived bladder matrix for the repair of endogenous brain tissue after stroke. They also found that only softer hydrogels that matched the mechanical properties of brain tissue could be used to promote neural tissue repair in the lesioned cavity. In addition, the shape adaptability of the hydrogel is another reason for its ability to efficiently promote nerve tissue repair. Different types of neurological tissue defects require hydrogels in different shapes for treatment. For example, stroke causes irregularly shaped neural cavities in brain tissue, which requires hydrogels to fill and repair in a matching shape. For spinal cord and peripheral nerve injuries, hydrogels in the form of tubes are more conducive to bridging and repair. Wang et al. (2020) reported an injectable silk-gel scaffold doped with carbon nanotubes for stroke treatment. The experiments showed that the scaffold could precisely match any irregularly shaped neural cavity after injection into the brain tissue, thus accelerating the growth of new neuronal networks into the cavity. Hamid et al. (2021) successfully developed a bionic tubular scaffold composed of polycaprolactone, alginate or gelatin methacrylate and loaded with embryoids to promote spinal cord tissue repair by 3D printing technology. Gao et al. (2023) reported a hybrid gelatin and hyaluronic acid hydrogel capable of modulating the immune microenvironment for helping damaged spinal cord tissue achieve complete repair. He et al. (2019) developed a hyaluronic acid-methylcellulose hydrogel modified with anti-inflammatory peptides and brain-derived neurotrophic factors. The hydrogel could promote functional recovery of the damaged spinal cord by modulating inflammatory cytokine levels and improving axonal regeneration. Liu et al. (2022) reported a dopamine-modified chitosan hydrogel. The hydrogel has excellent biocompatibility and antioxidant properties, which can improve the adverse microenvironment of the damaged spinal cord thus promoting the repair and regeneration of spinal cord tissue. Lingling Chen et al. (2022) designed a conductive hydrogel (MoS_2_/GO/PVA) based on graphene oxide, molybdenum sulfide, and polyvinyl alcohol. This kind of hydrogel has excellent electrical conductivity and anti-inflammatory activity, and can promote endogenous regeneration of spinal cord tissue and inhibit activation of glial cells at the site of injury, for which the hydrogel can restore the function of spinal cord tissue (Table 2).

**TABLE 2:** Applications of hydrogels for repair and regeneration of tissue defects.

Applications	Classification	Representative hydrogels
Cartilage tissue defects	articular cartilage defects	GelMA-AGA/VPA (Chen Z. et al., 2021); CS/PCL/SMSCs/TFNA (Li P. et al., 2021)
Bone tissue defects	bone defects; osteoporosis	BP/alginate/NIPAM (Wang X. E. et al., 2022); DACNC/CHI-C (Huang W. J. et al., 2021); ICPN/DHCP (Kuang et al., 2021)
Skin Wounds	infected wounds; burn wounds; diabetic wounds	Gel-DA/GG@Ag NPs (Zhang H. et al., 2021); CCOD-MgO (Tang et al., 2021); CS-AT-Exo (Wang K. et al., 2022)
Muscle tissue defects	skeletal muscle defects; myocardial defect	MAA-collagen (Carleton et al., 2021); P(CS-CA-NIPAM) (Jiang et al., 2022)
Nerve tissue defects	brain injury; spinal cord injury	Cur-TM/PC (Qian et al., 2021); MoS_2_/GO/PVA (Chen L. et al., 2022)

## 4 Discussion and prospects

This paper summarises and discusses four prominent advantages of hydrogels and their application and progress in the repair and regeneration of tissue defects. We believe that with continued development and practice, functional hydrogels will change the paradigm of tissue defect treatment and become the most competitive material in tissue engineering for use in the clinical setting. Transplantation, with limitations such as surgical complexity, immune rejection and lack of donor tissue, will be replaced by hydrogel therapy, which is safe, stable, consistently efficient, easy to control, easy to use and highly spatiotemporally selective with a low invasive load. At the same time, hydrogels, as intelligent delivery systems capable of releasing therapeutic reagents on demand, will allow individualised treatment based on patient specificity and tissue defect specificity, thus greatly improving treatment efficiency and saving economic costs. Despite the obvious advantages of hydrogels in the field of tissue regeneration, many challenges still need to be overcome before hydrogels can be used in clinical applications. First, the function of hydrogels is currently being studied mainly through *in vitro* cellular and *in vivo* animal experiments. While these studies are easy to monitor, convenient and reproducible, they still do not provide a true and accurate picture of the long-term effects of hydrogels in the regeneration of human tissue defects. Second, the specific mechanisms and signalling pathways by which many hydrogels interact with cells have not been fully investigated, which is one of the key reasons why hydrogels are still difficult to use in the clinic. Finally, the properties of hydrogels can vary depending on their material composition, concentration, synthesis strategy, manufacturing process and cross-linking method, for which before hydrogels can be used on a large scale in the clinical setting, it is essential that they are screened and optimised to construct a library of materials to standardise these factors influencing hydrogel performance. In conclusion, hydrogels have outstanding advantages in the repair and regeneration of tissue defects and are likely to change the treatment paradigm of tissue defects, but there is still a long way to go, and we need to continue our research and exploration.
